# Supplementary material for: Allometric-like scaling of AAV gene therapy for systemic protein delivery
Source: Mol Ther Methods Clin Dev. 2022 Oct 21;27:368–79. doi: 10.1016/j.omtm.2022.10.011 (PMC9641010; doi:10.1016/j.omtm.2022.10.011)
Supplement: Document S1. Figures S1 and S2 and Table S1 [file mmc1.pdf]

OMTM, Volume *27*

Supplemental information

Allometric-like scaling of AAV gene therapy for systemic protein delivery

Alexandra Burr, Patrick Erickson, Raphaela Bento, Kariman Shama, Charles Roth, and Biju Parekkadan

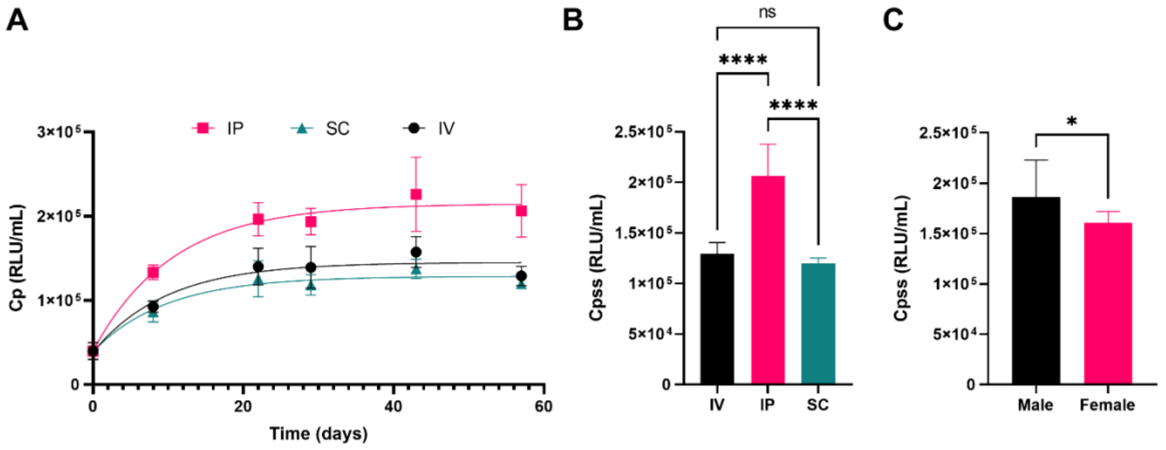

Figure S1. GLuc concentration varies with systemic administration route. (A) Concentration of GLuc in the plasma (Cp) over time (RLU/mL) for each administration route: IV = intravenous, IP = intraperitoneal, SC = subcutaneous. Data points show are the mean ± SD for replicates (n=8, 4 male/4 female, per group). Solid lines represent curve-fit for an exponential plateau equation to estimate steady-state. (B) Steady-state concentration (RLU/mL) as determined by the curve-fit is shown for each administration route. (C) Comparison of male and female concentrations for all routes of administration. Bars represent mean ± SD for replicates. NS = non-significant, *p<0.05, **** p<0.0001.

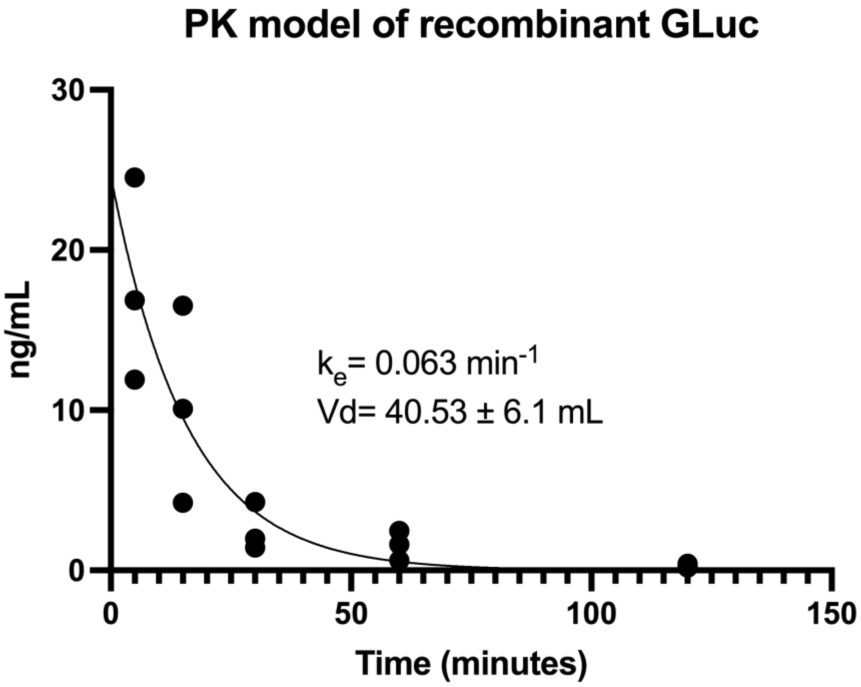

Figure S2. A single dose kinetic study with 1ug of the recombinant Gluc protein injected intravenously (n=3 mice/timepoint). Parameters for GLuc were determined by fitting the data to Equation 3 and extracting values of the elimination constant and the volume of distribution.

Table S1. Study information for data sets used for literature mining study

Publication	Serotype	Promoter / Transgene	# of doses tested	# of mice	Route of Administration	Sex, Strain	Timepoint for dose-response
Nathwani et al, 2009	scAAV2/8	LP1-hFIX	4	5-8	tail vein IV	male C57BL/6	57 days
	scAAV2/9						
	scAAV2/7						
	scAAV2/rh10						
Nathwani et al, 2006	scAAV2/8	LP1-hFIXco	4	4	tail vein IV	male C57BL/6	42 days
	ssAAV2/8	HCR-hAAT-hFIX	6				
Davidoff et al, 2005	ssAAV5/5	HCR-hAAT-hFIX	8	4	tail vein IV	male C57BL/6	116 days
	ssAAV2/8		6				
Grimm et al, 2003	ssAAV2/2	HCR-hAAT-hFIX	6	4-5	portal vein	C57BL/6	42 days
	ssAAV2/1		5				
	ssAAV2/6		7				
Nakai et al, 2002	ssAAV2/2	HCR-hAAT-hFIX	9	5	portal vein	female C57BL/6, male C57BL/6 rag-1	70 days
Nakai et al, 2005	ssAAV2/1	EF1α-nlslacZ	4	3-6	portal vein	male C57BL/6	42 days
	ssAAV2/2						
	ssAAV2/8						

Abbreviation: Ss: single-stranded genome, sc: self-complementary stranded genome
