# Supplementary material for: Allometric-like scaling of AAV gene therapy for systemic protein delivery
Source: Mol Ther Methods Clin Dev. 2022 Oct 21;27:368–79. doi: 10.1016/j.omtm.2022.10.011 (PMC9641010; doi:10.1016/j.omtm.2022.10.011)
Supplement: Document S2. Article plus supplemental information [file mmc2.pdf]

Molecular Therapy
Methods & Clinical Development
Original Article

Allometric-like scaling of AAV gene therapy for systemic protein delivery

Alexandra Burr,[1] Patrick Erickson,[2] Raphaela Bento,[1] Kariman Shama,[1] Charles Roth,[1,2] and Biju Parekkadan[1]

[1]Department of Biomedical Engineering, Rutgers University, Piscataway, NJ, USA; [2]Department of Chemical and Biochemical Engineering, Rutgers University, Piscataway, NJ, USA

The use of adeno-associated virus (AAV) as a gene delivery vehicle for secreted peptide therapeutics can enable a new approach to durably manage chronic protein insufficiencies in patients. Yet, dosing of AAVs have been largely empirical to date. In this report, we explore the dose-response relationship of AAVs encoding a secreted luciferase reporter to establish a mathematical model that can be used to predict steady-state protein concentrations in mice based on steady-state secretion rates *in vitro*. Upon intravenous administration of AAV doses that scaled multiple logs, steady-state plasma concentrations of a secreted reporter protein were fit with a hyperbolic dose-response equation. Parameters for the hyperbolic model were extracted from the data and compared with create scaling factors that related *in vitro* protein secretion rates to *in vivo* steady-state plasma concentrations. Parathyroid hormone expressed by AAV was then used as a bioactive candidate and validated that the model, with scaling factors, could predict the plasma hormone concentrations in mice. In total, this model system confirmed that plasma steady-state concentrations of secreted proteins expressed by AAVs can be guided by *in vitro* kinetic secretion data laying groundwork for future customization and model-informed dose justification for AAV candidates.

INTRODUCTION

Protein-based therapeutics are one of the most successful and widely used classes of pharmaceuticals, including a broad range of recombinant proteins and peptides with multiple applications. Monoclonal antibodies and hormone therapy are examples of highly successful and evolving classes of protein therapeutics in industry.[1,2] Nonetheless, the complex nature of these molecules, poor stability, and low bioavailability represent major drawbacks to their use in the clinic.[3,4] Depending on the type and stage of the disease, these proteins may need to be administered multiple times, periodically. Many peptide-approved formulations are commercialized in suspension form for self-administration, relying on the compliance of the patient to the treatment. It has been reported that adherence is typically not sustained for over 6 months of treatment, especially in cases of chronic conditions.[5] These limitations highlight the importance of alternative approaches to deliver protein therapeutics with sustained and controlled release for longer durations.

Gene therapeutics have been designed to express therapeutic proteins for long-term molecular therapy. Numerous viral and non-viral vectors have been used to introduce genetic material into target cells, providing protein expression for prolonged periods of time without patient interaction. This emerging field represents a viable solution to bypass the need of patients relying on daily drugs, providing them with a self-sustaining solution. Notably, adeno-associated viruses (AAVs) have been extensively explored as gene delivery vehicles for several human diseases, representing a relevant therapeutic modality. AAV gene therapy has shown clinical efficacy over the years, enabling site-specific targeting of distinct disease conditions.[6–9] Over 50 clinical trials utilizing AAVs as delivery vectors for secreted peptide therapeutics are currently in progress, covering a wide range of applications from hemophilia, Parkinson's disease, muscular dystrophy, to Alzheimer's disease and choroideremia.[4,10] Translation of AAV gene therapy candidates is largely based on empirical studies to determine a favorable pharmacokinetic/pharmacodynamic relationship, although several factors can ultimately determine a protein concentration in the bloodstream and can be critical to optimize. For example, viral serotypes have varied tissue tropisms and expression levels that impact secreted protein levels in mouse studies.[11,12] Rational design-directed capsid engineering to alter vector serotypes is one emerging approach to create new variants with improved pharmacological properties.[13] Furthermore, the promoter and transgene designed into AAV can greatly affect a secreted protein concentration in the blood.[14] Alterations to promoter strength as well as transgene variants are often considered to optimize the secretion of the therapeutic protein for a given indication.[15] The screening process takes place *in vitro* and multiple vector candidates may be tested in animal models once hits are identified, or the *in vitro* study is skipped entirely.[16–18] This reliance on *in vivo* models is generally attributed to the poor translation of transduction efficiency between relevant human cell lines and mouse models.[19] As such, a mathematical model correlating the dose of viral genomes (vg) to the steady-state blood concentrations of secreted therapeutic proteins could have a great impact in the field, serving as a model-informed approach to better predict *in vivo* results and minimize the number of animals needed for optimization of a given vector.

Received 5 August 2022; accepted 18 October 2022;
https://doi.org/10.1016/j.omtm.2022.10.011.

Correspondence: Biju Parekkadan, Department of Biomedical Engineering, Rutgers University, Piscataway, NJ, USA.
E-mail: biju.parekkadan@rutgers.edu

 © 2022 The Author(s).
This is an open access article under the CC BY license (http://creativecommons.org/licenses/by/4.0/).

The pharmacological concept of allometric scaling from one model organism to another, pioneered in small-molecule development, is just beginning to emerge for gene therapeutics.[20–23] In 2018, the FDA launched a Model-Informed Drug Development Pilot Program to "facilitate the development and application of exposure-based, biological, and statistical models" for cell and gene therapies in development.[24] This program encourages researchers to assess model risk and develop predictive metrics that inform dosing for these drugs. In a recent study and commentary, authors looked at the allometric scaling relationship for AAV-based FIX hemophilia treatment from mice to dogs to non-human primates, to humans. They identified a correlation between them that is the inverse of what is seen in traditional pharmaceuticals; where the effect typically increases with body weight, AAV-based treatment results in decreased effect with increased body weight.[25,26] The correlation is found in terms of a novel parameter declared as a gene efficiency factor, which utilizes the steady-state concentration reached in the blood as well as the dose. This was the first ever reported finding for developing an allometric scaling relationship for AAV gene therapy and showed a need to increasingly explore this field. Importantly, in addition to understanding the translation between different model organisms as explored in these initial reports, a better understanding of translational *in-vitro*-to-*in-vivo* correlations would have a broad impact on preclinical development.

In this report, we developed a mathematical relationship between *in vitro* protein secretion dose-response data and *in vivo* steady-state blood concentration dose-response data using AAV2 expressing a secreted luciferase reporter as a model system. Our results showed that *in vivo* dose-response curves could be fit to a Hill equation with a strong correlation of fit. This model was further validated using seven additional literature datasets that curve fit precisely. By extracting equation parameters from these historical studies, we also drew comparisons between the 50% effective dose (EC_{50}) for different vectors and showed the importance of using a wide dose span when developing a model. As liver tissue showed highest vector genomes, we developed a bioassay using a human hepatocellular carcinoma line (HepG2 cells) cultured in a perfusion system with kinetic sample collection to observe protein secretion rate as a simplistic model for *in vivo* blood concentration levels. A hyperbolic relationship between viral dose and steady-state cell secretion rate was also observed in this HepG2 assay. Scaling factors were applied to connect the *in vitro* and *in vivo* datasets. We tested the predictivity of this allometric scaling approach using a therapeutic peptide, parathyroid hormone (PTH), and showed that dose response in mice can be predicted from our hepatocyte culture model with great accuracy. The model herein described reports a novel method to predict the failure or success of achieving therapeutic concentrations following *in vitro* vector optimization studies.

RESULTS

In vivo pharmacokinetic studies of secreted GLuc from AAV2 in mice

The kinetics of protein concentration in the blood can be altered by many variables related to the AAV vector. However, AAV-mediated protein concentration has been shown to reach a steady-state within 4 weeks of injection and is a consistent phenomenon.[4] To ensure this phenomenon was consistent with our vector, we performed a kinetic study following administration of AAV2-EF1α-GLuc at two doses (2.5×10^9 and 1×10^{10} vg) in mice. The concentration of GLuc (*Gaussia princeps* luciferase) was measured in the plasma of mice over time starting at 3 days and continuing until 8 weeks post-injection. As shown in Figure 1A, the concentration increased in the first 3 weeks and began to reach a steady-state concentration thereafter. After fitting Equation 4 to the data, the steady-state concentration (C_{pss}) of GLuc was estimated to be 2.7 and 7.3 ng/mL for the low and high dose, respectively. The GLuc levels reached ~95% of their steady-state values within 3 weeks. C_{pss} was impacted by administration routes and gender (Figure S1). For consistency, male mice and intravenous administration route were used for all subsequent studies. With the time to reach steady-state concentration determined, a dose-response study was performed using AAV-EF1α-GLuc, in which the concentration of GLuc in the blood was measured 3 and 5 weeks post-injection. As shown in Figure 1B, the average GLuc concentration increased with increasing AAV dose (vg/mouse). Doses lower than 10^9 vg/mouse resulted in GLuc concentrations below the detection limit of the assay.

The tissue-specific vg content after intravenous administration AAV2 was also measured using qPCR. Five tissue types were measured and, as shown in Figure 1C, significantly more AAV genomes were present within liver tissue than spleen, kidney, heart, or lung tissue. The concentration of vg in the liver tissue was measured to be over 10 times higher than any other tissue type measured. Combined with the fact that the liver has significantly greater mass than the other organs, we concluded that a systemic administration route of AAV2 resulted in primarily liver cell transduction, which was considered as the primary organ of protein production.

Fitting *in vivo* pharmacokinetic data using a compartmental model

The mechanisms that determine the ultimate plasma concentration of a transgenic protein for a given AAV dose are complex; the secretion rate is affected by initial distribution throughout the body, binding to cells, translocation, unpackaging, expression, and secretion, while the volume of distribution and elimination rate are determined by the protein and the animal (Figure 2A). Despite this, we found the dose-response curves were well-fit by the E_{max} model with a Hill coefficient set to 1 denoting no cooperative binding, an equation used to model many other biological dose-response relationships.

Equation 3 (see methods) was fit to data from the single dose pharmacokinetic study for GLuc (Figure S2) to yield the values $V_d = 40.5$ mL and $k_e = 95.0$ day^{-1}. Following the *in vivo* dose ranging studies, V_d and k_e were used in Equation 5 to convert the measured C_{pss} values to R_{ss} values (Figure 2B). These were then plotted against their corresponding AAV doses (Figure 2C) and fit with the E_{max} model equation (Equation 6) with an assumed Hill coefficient of 1. A Hill coefficient of 1 suggests that the dose-response relationship may be governed by Michaelis-Menten binding of viral particles to target

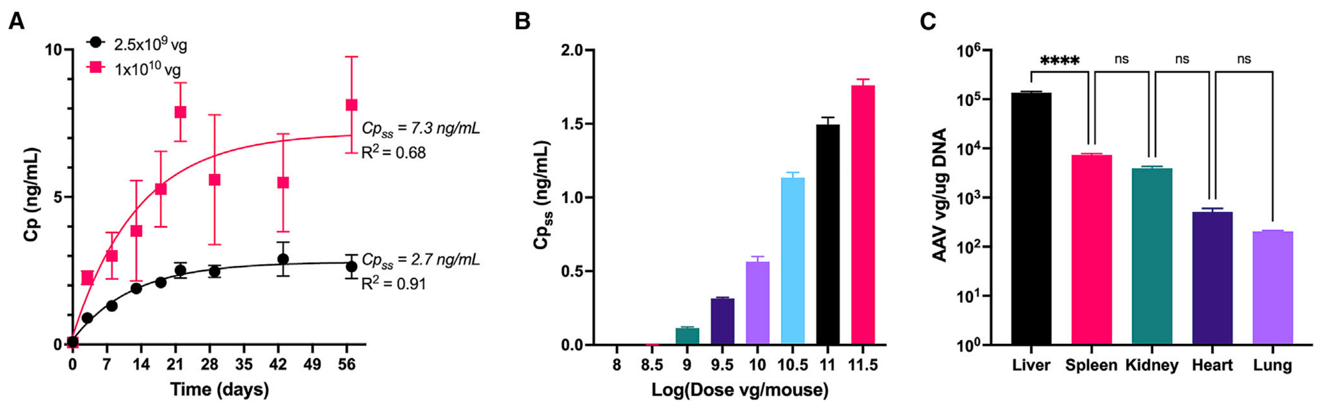

Figure 1. *In vivo* dose response of a secreted reporter protein at steady-state concentrations

(A) Example of plasma concentration (Cp ng/mL) of GLuc over time following an intravenous AAV injection. Data points represent mean ± SD for n = 4 mice. Solid lines show the fit increasing exponential decay model (Equation 4). Cpss is the steady-state plasma concentration found using the model fit. (B) Plasma concentrations of GLuc at steady state for the indicated AAV dose. Bars represent mean ± SD for n = 3 mice for each dose. (C) Viral genome quantity as measured by qPCR for various tissues from animals injected with $10^{11.5}$ vg. Bars represent mean ± SD for n = 3 mice. n.s., not significant; ****p < 0.0001.

receptors. Due to the Hill value equaling 1, it was dropped from the final scaling equation. The fit parameter values shown in Figure 2D suggest that the maximum R_{ss} that can be achieved using the given AAV is R_{max} = 46.35 ng/h and that the AAV dose resulting in the half-maximum response is $EC_{50} = 10^{10.68}$ vg. The E_{max} equation fit the data well, with an R^2 of 0.991.

Literature dose-response model

Given that the relationship between dose and secretion rate *in vivo* is hyperbolic, we sought to determine if the same relationship can be used to represent literature data that measured protein secretion or expression at steady state from AAV. Criteria were established for inclusion of studies in this retrospective analysis. Only datasets that tested at least four doses and included a figure that showed a dose response were used in the literature mining. In addition, data had to be from mouse samples after systemic AAV administration. Due to the limited availability of data, we did not constrain the serotype used. Time dependency was accounted for by only using concentrations that could be assumed to be at steady state which was set at greater than 28 days post-injection.

Using such studies, we determined the correlation of fit for a hyperbolic dose curve to represent the data. A supplementary table showing the publications and datasets is shown in Table S1. Six publications were used, which each contained at least two dose-response curves. For each dataset, Web plot Digitizer software was used to extract the data points from dose-response figures by calibrating axes. The extracted data (dose, Cpss) were plotted, and curve fit to a three-parameter logarithmic equation where the Hill coefficient was constrained to 1 and the base value was set to 0. Figure 3A shows the correlation between hyperbolic dose-response curve fit for seven different studies using AAV to measure protein expression or secretion at steady state with a total of 72 mice.[27–32] The overall correlation was greater than 0.98 (R^2). From these curve fits, parameters shown previously, such as the dose as half-maximal response (EC_{50}), were extracted and compared across studies. The average EC_{50} value for each study falls in the range of 10^9 and $10^{11.5}$ vg/mouse and has an overall mean of $10^{10.58}$ vg/mouse (Figure 3B). Finally, to investigate the importance of measuring a wide dose span when created a hyperbolic dose-response curve model, the correlation based on sum of square errors was plotted as a function of the dose span. As shown in Figure 3C, there is a weak negative correlation between dose span and sum of squares for the datasets used in this model, which suggests that using a larger dose span reduces the error of the model equation.

In vitro HepG2 transduction and kinetic GLuc secretion study

Studies were conducted to determine whether a similar hyperbolic relationship existed between viral dose and steady-state secretion rate *in vitro* as was found *in vivo*. A continuous perfusion bioreactor culture system integrated with downstream fraction collection was used to measure GLuc secretion rate from transduced cells as a model for *in vivo* tissue release of GLuc into the bloodstream (Figure 4A). Using a human hepatocellular carcinoma cell line (HepG2) *in vitro* as a model for liver parenchymal cell transduction, a dose-response curve was derived. Cells were perfused for 18 h, starting at 72 h post-AAV2 transduction. Medium was collected as fractions hourly, and the average GLuc values from all fractions after 7 h of perfusion were averaged. The GLuc secretion rate for each AAV dose was compared with the transduction efficiency of the cells in the corresponding culture as measured by GFP transduction. Figure 4B shows the increased GLuc concentration and corresponding transduction efficiency for cells transduced with increased dose.

To determine if the *in vitro* dose-response curve was similar to that observed *in vivo*, the secretion rates were calculated from GLuc concentrations using the perfusion rate of the system. Secretion rate was plotted as a function of the log(dose) in Figure 4C. The secretion rate

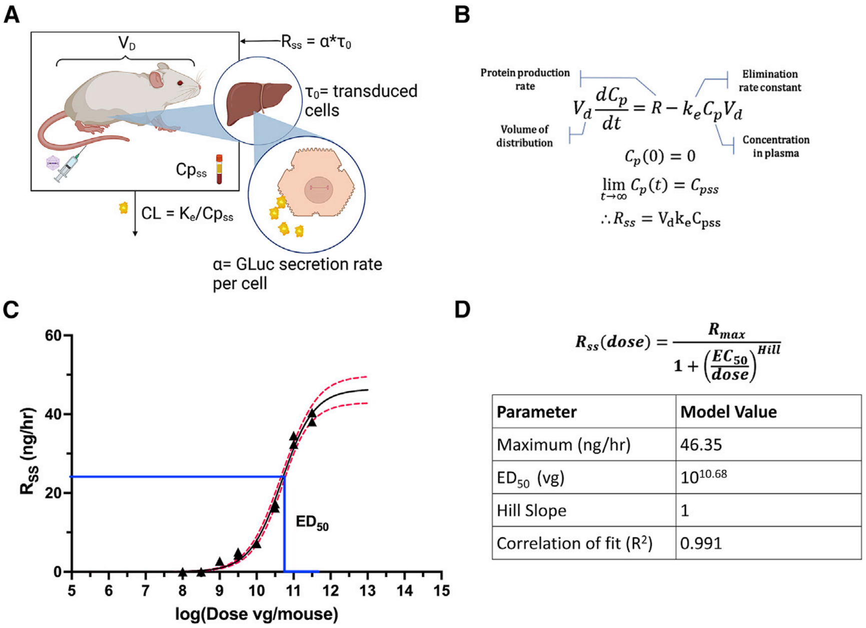

$$R_{ss}(dose) = \frac{R_{max}}{1 + \left(\frac{EC_{50}}{dose}\right)^{Hill}}$$

Parameter	Model Value
Maximum (ng/hr)	46.35
ED_{50} (vg)	$10^{10.68}$
Hill Slope	1
Correlation of fit (R^2)	0.991

Figure 2. Modeling the phenomenon *in vivo*

(A) The mechanistic relationship between the AAV dose and the ultimate transgene plasma concentration is highly complex and depends on overall transduction and the physiology of the animal. (B) Derivation of relationship between the steady-state concentration and secretion rate from the overall mass balance. (C) Dose response of steady-state rate *in vivo*. Black triangles represent mean ± SD for n = 3 mice. Black line: Hill equation dose-response curve fit (Equation 6). Red dotted line: 95% confidence interval. (D) Hill equation and extracted model parameters.

exhibited a classic dose-response relationship in which lower doses yielded similar rates, middle-range doses had larger separation, and the highest doses approach near maximal rates. Using the E_{max} equation to fit the data points yielded a correlation of fit of 0.973, showing a strong correlation for this relationship also observed *in vivo*.

Parameter scaling from *in vitro* to *in vivo*

The main vision of this work is to establish a relationship that enables the dose response of a novel AAV measured in cell culture to predict the dose response of that AAV in a mouse model. Parameters from *in vitro* and *in vivo* curve fitting were extracted and compared with understood scaling factors that could be incorporated into the *in vitro* hyperbolic equation to yield the *in vivo* equation. Figure 5A shows a conceptual representation of the datasets. When comparing the two curves, the dose at half-maximal rate is lower in mice than in HepG2 cells and the maximal secretion rate is much higher.

Figure 5B shows the scaling equation along with the parameter values for both systems. As shown in the table, the *in vitro* maximal rate (R_{ss}) of 0.1585 ng/h was 290 times smaller than the *in vivo* value of 46.35 ng/h. The log(dose) at half-maximal rate (EC_{50}) in mice was 10.68 which was 1.1 log order of magnitude higher than observed *in vitro*.

Predictivity testing

In theory, *in vitro* dose-response data could now be scaled using these factors to predict a targeted steady-state plasma concentration *in vivo*. We tested this hypothesis to determine the predictive power of this scaling model using another transgene. For this study, we tested an AAV2 vector with an EF1α-PTH transgene. PTH encodes for full-length human PTH and represents a clinically relevant therapeutic protein. In addition, PTH is of interest because PTH deficiency is currently treated with an injectable recombinant hormone due its short half-life.

AAV2-EF1α-PTH was used to transduce HepG2 cells at varying doses and the data were compared with the previously obtained GLuc data. Surprisingly, the steady-state secretion rates were almost identical between the two proteins at the same doses (Figure 6A). While R_{ss} remained nearly the same between the two proteins, the half-life of PTH *in vivo* is 4 min, which is significantly less than the 11 min for GLuc. This model assumes that the volume of distribution is the same for GLuc and PTH. Given these variables, the steady-state equation included in Figure 6B was used to calculate the concentration based on secretion rate as a function of dose. This equation now accounted for scaling from an *in vitro* system to an *in vivo* system (as determined previously for GLuc) as well PTH-specific parameters. Using this scaled equation, a predicted PTH C_{pss} dose-response curve was plotted for PTH, as shown in Figure 6C. Then, we plotted the actual data from animals receiving two different doses of AAV2-EF1α-PTH. As shown, the predicted values were very close to the actual value without introducing any additional parameters. The sum of squares error between the actual and predicted data was 4.034 pg/mL. These results support the utility of these scaling factors for other secreted transgenes that are delivered by AAV2.

DISCUSSION

Gene therapeutics as an alternative modality for delivery and sustained release of secreted factors have been increasingly explored in pre-clinical studies and clinical trials. AAV has become one of the most favored and widely used genetic material delivery systems, with promising results in the clinic. However, the field still has a wide variable space required to optimize gene products for clinical use. To that end, several factors must be considered, such as cellular heterogeneity, diverse cellular trafficking across different AAV serotypes, *in-vitro*-to-*in-vivo* correlation and interspecies extrapolation.[4,33–35] Model informed drug development has gained attention in almost all therapeutic areas in the last two decades.[4,36] Yet, gene-based therapeutic modalities have fallen behind these developments, due to major limitations in modeling transduction efficiency *in vivo*.

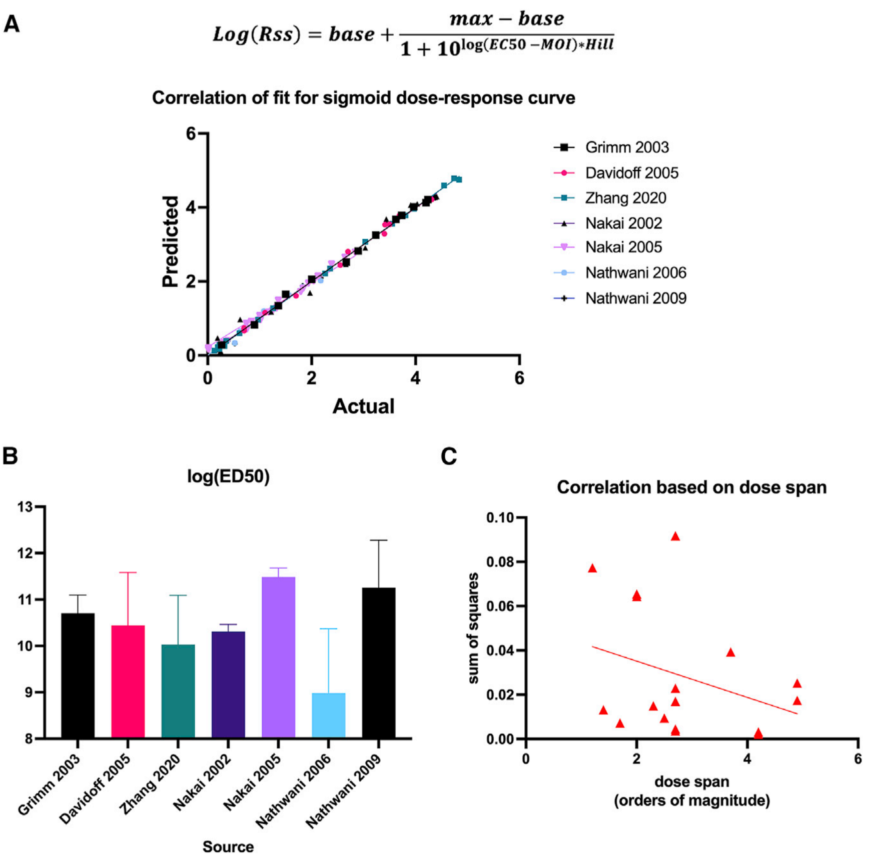

Figure 3. Literature data support sigmoid dose-response models

(A) Correlation of fit for seven studies with n > 4 mice/dose [27–32] showing actual data versus predicted data with a sigmoid dose-response curve fit. (B) Parameter results for the log(EC_{50}) from each study. Bars represent mean ± SD for all experiments in each study (average no. of experiments for each study = 2). (C) Correlation parameter (SSE) as a function of the dose span used for each experiment.

The study herein described developed a novel pharmacokinetic approach to guide dose selection of secreted proteins from AAV2 vectors using a secreted luciferase reporter. *In vivo* steady-state plasma concentrations of GLuc were found to be strongly fit by an E_{max} model curve as were several other literature studies that explored secreted protein therapeutics from AAVs.[27–32] This model sets the Hill coefficient to 1 to minimize parameters in the model and assumes that transduction events are independent, that is, viruses do not cooperate to achieve transduction/expression nor does transduction of one cell inhibit the transduction and expression of neighboring cells. When left unconstrained, it was interesting that the Hill coefficient remained constant for both systems when using the same vector of ~0.9 (data not shown), which is fairly close to 1, although this suggests that there may be a small amount of negative cooperativity, presumably due to cellular immunity. While there are many variables that can influence the secretion of a protein from AAV transduction, we focused on a narrow set of parameters to elucidate dose-response curves in HepG2 cells and mice using an AAV2 vector encoding for GLuc secretion with a strong EF1α promoter. Parameter-scaling criteria were then determined between *in vitro* cellular expression data and *in vivo* plasma concentrations. These scaling factors were successfully deployed to predict the *in vivo* concentration of an unknown protein, PTH, using the same vector. There were several important learnings and limitations of this research, which are further discussed.

It is known from the literature that it takes several weeks for *in vivo* expression following AAV administration to reach its maximal value. Likewise, expression will eventually decline as the gene, which is not fully integrated into the chromosomes, is lost. For this work, we focus on the period in which expression is maintained at a reasonable steady state. While the pharmacokinetic model takes into account the onset time of expression, decay or loss of expression is an unsteady-state phenomenon that is not determined by our steady-state profile. At this point, we have not speculated on how to predict the length of steady state or the time until decay. To do this, long-term studies that examine the time to decay as a function of dose and compare this with the steady-state signal would need to be performed. It is likely that the correspondence between *in vitro* and *in vivo* dosing would still hold, although the scaling of expression would then be time dependent.

Because of the broad variations in AAV potency, the goal of this model is to establish an *in vitro* procedure to be performed by researchers, allowing them to use experimental data to predict outcomes in mice. To control for serotype, AAV2 was used throughout this work. Importantly, it has been shown that liver is the primary tissue transduced regardless of viral serotype, but the level of transduction may vary depending on the AAV subtype.[37] Translation of the model to other serotypes may also be limited due to transduction disparities between human and murine cells. For example, AAV8 exhibits much lower transduction efficiency in HepG2 cells, while transducing mouse hepatocytes much more efficiently than AAV2. Hence, serotype-dependent relationships could be further investigated in future studies where the scaling factor between *in vitro* and *in vivo* is serotype dependent.

This study is the first to examine the dose response over many orders of magnitude by intravenous administration of AAVs. Publications from the early 2000s focused on a small dose range, while those published more recently have expanded the dose range across multiple orders of magnitude.[6,29–31] At the high dose, there was more between-subject variability than is commonly seen with AAV expression, suggesting the importance of employing high-power

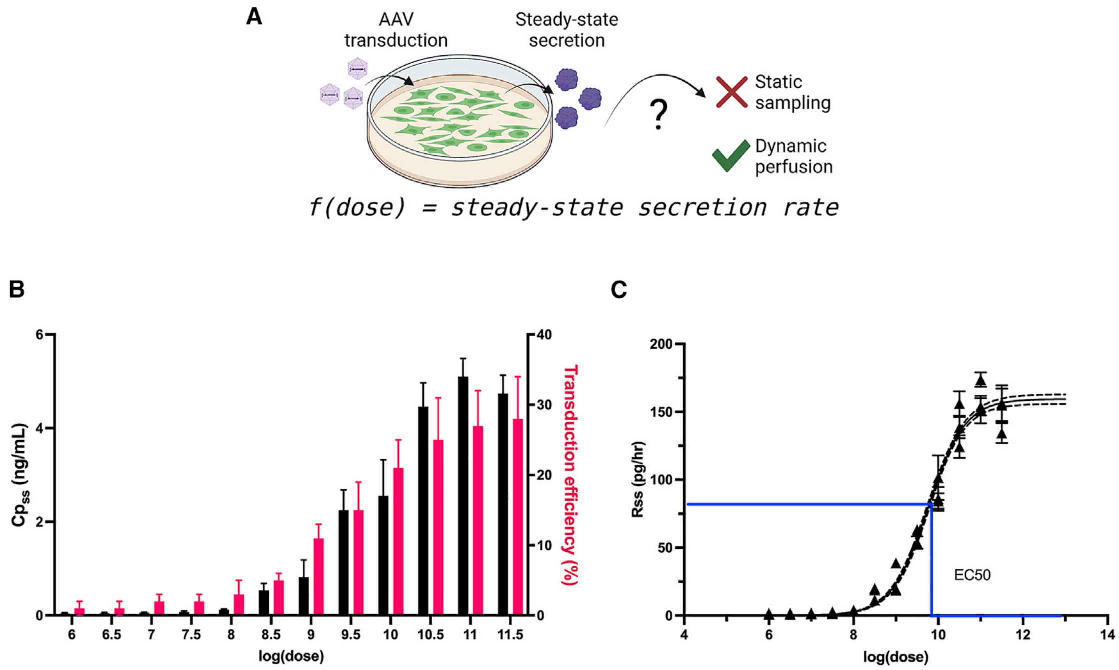

Figure 4. *In vitro* dose response measured using a dynamic culture system

(A) *In vitro* perfusion culture system allows for dynamic measurements of transgenic protein secretion rate from AAV-transduced cells. (B) *In vitro* concentration of GLuc (black) and transduction efficiency as measured by GFP+ cell number (pink) as a function of dose. (C) Calculated secretion rate as a function of dose. Black curve: sigmoid dose-response model Hill equation. Dotted line: 95% confidence interval. Data points show ± SD for n = 7 steady-state concentrations per well. Blue line: EC_{50}.

studies and multiple time points to have a more precise estimate of steady-state blood concentration. Meta-analysis of other trials also confirmed a weak negative correlation between dose span and prediction error, which further supports the consideration to use a larger dose span to reduce the error of model equation. In addition, literature data evaluation showed that the same curve fitting can be used to model dose response from other vectors of various serotypes, promoters, and transgenes. These findings are important not only for predictive concentrations of a therapeutic peptide, but to the broader understanding of AAV vector cell entry. Other tissue-specific relationships can be evaluated to better understand if this is unique to the liver or simply the receptor type that is the primary mechanism on hepatocytes for AAV transduction. Several other findings were also reproduced using our model system, such as a sex difference in expression[38] and differences in blood concentrations depending on the administration route—the intraperitoneal groups reached steady-state concentrations almost 2-fold higher than the subcutaneous group, similarly to previous reports using neonatal mice transduced with AAV serotype 5.[39] These findings highlight the importance of keeping the route of administration consistent when comparing expression or concentration data for a given vector.[40,41] These are areas of independent investigation that could be further adapted in the model, broadening our current mechanistic understanding.

Using a hepatocellular carcinoma cell line as an *in vitro* model of liver parenchymal cell transduction, the feasibility of using an *in vitro* model to predict *in vivo* dose response was assessed. Tissue analysis of mouse livers was utilized to assess the transduction efficiency that can be used to estimate the apparent multiplicity of infection (MOI) of the dose used. MOIs used for transducing the HepG2 cell line typically range from 20,000 to over 200,000 vg/cell and fail to achieve over 60% transduction efficiency. Literature data have shown that there is a limit to transduction efficiency in hepatocytes, so our hypothesis was that the secretion rate would reach a plateau at the highest MOIs. In addition, given the data showing that HepG2 cells must be transduced at higher MOIs than some other cell types to measure expression or secretion, we also hypothesized that there would be a threshold MOI for measurable secretion that is non-zero. To test the full range of HepG2 secretion rates *in vitro*, cells were transduced with a broader range of doses equivalent to MOIs ranging from 10 to 10,000,000 vg/cell to resolve a complete dose-response curve. This was the first reported hyperbolic dose-response curve from *in vitro* secretion after AAV transduction and it exhibited a very strong correlation. While trends with apparently hyperbolic relationships could be visualized from previous studies, it was interesting to see the strong correlation between the transduction efficiency and the secretion rate, which supports the idea that viral uptake is a limiting factor in transduction efficiency. The baseline expression can be set to zero and without constraints, the model found a value approximate to zero. Mechanistically, this suggests that there is a threshold dose to achieve sufficient transduction for expression because the

A

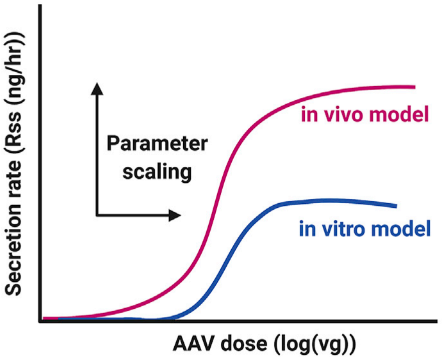

B

$$R_{ss,vivo}(dose) = \frac{290 * R_{max,vitro}}{1 + \frac{EC_{50,vitro} + 1.1}{dose}}$$

Parameter	*In vitro*	*In vivo*
R_{max} (ng/hr)	0.1595	46.35
EC_{50} (vg)	$10^{9.762}$	$10^{10.68}$
R^2	0.9725	0.991

Figure 5. Applying a scaling function to create an applicable model

(A) Data from cells and mouse dose response can be used to derive a scaling relationship between the two system by identifying differences in the maximum secretion rates and the ED_{50}. (B) Using model curves, the resulting scaling equation is shown for *in vivo* rate as a function of the cellular parameters. The table shows values from both models used to calculate scaling equation.

non-zero secretion rates from the lower doses were well above the detection limit of our assay. It is important to note that, when using this system, the sensitivity of the assay used to measure dose response is critical, and larger culture systems may be needed to raise the concentrations to measurable ranges. For instance, GLuc can be detected with as little as 0.1 pg/mL, while PTH exhibits a higher detection limit of 25 pg/mL.

When applying the model trained on data for GLuc to expression of PTH, we found that their *in vitro* expression rates were quite similar, and that the model successfully predicted the *in vivo* expression by accounting for the differing pharmacokinetic behavior of PTH. While the *in vivo* half-life of GLuc is around 11 min, the circulating half-life of native PTH is only about 4 min.[42,43] The similar *in vitro* expression rates are likely due to the fact that GLuc and PTH are both relatively small, globular, and of similar size: PTH is 14.1 kDa large and GLuc is 18.2 kDa. Future investigation should consider proteins of drastically different sizes to determine how that impacts secretion rate. In addition, another factor that was not considered in our model was the variable immune response that can occur based on the transgene. An immune response can significantly impact the longevity of expression and the stable concentration achieved.[4]

Finally, the scaling relationship between the models can be interpreted as the difference in total cells as well as the maximum transduction efficiency of those cells. The level of expression should be the number of cells transduced multiplied by the specific expression per cell. The *in vitro* system here utilized was fixed with 200,000 cells per well, representing the size of the system. Conversely, in the *in vivo* model, neither the number of cells nor what were transduced were definitively measured. This difference in cell number is likely reflected in the model through the higher maximum secretion rate. A mouse hepatocyte *in vivo* likely has a different rate of expression for the transgene, even under a constitutive promoter, compared with human hepatoma cells. The second scaling factor that shifted the EC_{50} to the right likely accounted for this. Finally, the findings from this study lay the foundation for further investigations using larger animal models. Scaling the approach to larger models will be critical to create pharmacokinetic understanding that is relevant to human dosing. Just as with traditional allometric scaling models, gene therapy will need to be characterized to understand the efficiency loss at scale. Future investigation of such models can be used to evaluate safety factors and tolerability studies for a given protein or molecule.

In conclusion, this report described the development of a novel pharmacokinetic model of secreted protein rates from AAV vectors and demonstrated correlation of the model to data extracted from the literature. Furthermore, the predictivity of our model was demonstrated by using an additional protein within the same AAV vector. We expect that, when applied to a different serotype, the same approach would be expected to work but the scaling parameters would have to be re-estimated with a model gene, such as GLuc. Further investigation into AAV expression at tissue level will be key to understanding how the system can be perturbed for increased potency. Altogether, these studies provide a framework for scaling of *in vitro* studies of AAV-driven secreted proteins to target specific *in vivo* blood concentrations.

METHODS

Cell culture and mice

HepG2 human hepatocellular carcinoma cells (ATCC) were cultured in EMEM (Millipore-Sigma) supplemented with 10% FBS (Gibco) and 1% antibiotic/antifungal v/v (Thermo Fisher). HEK293T embryonic kidney cells (ATCC) were cultured in DMEM F/12 supplemented with 10% FBS and 1% antibiotic/antifungal. All cells were incubated in 5% CO_2 at 37°C.

C57BL/6J mice between 8 and 10 weeks old were used for all studies (Jackson Laboratories). Animals were housed at no more than four mice per cage and were allowed food and water *ad libitum* throughout the duration of the study. Within 1 week of arrival, mice were placed randomly into groups and identified used ear punches. For intravenous injection and tail bleeds, a tail vein restrainer was used. For euthanasia, animals were placed in a CO_2 chamber for 5 min and cardiac puncture was performed immediately to collect blood samples into heparinized blood tubes. Tissue extraction was done after the carotid artery was cut and 10 mL of ice-cold saline was perfused through the system by injection into the left ventricle. All animal work was performed in accordance with the ethical standards of the Institutional Animal Care and Use Committee (IACUC).

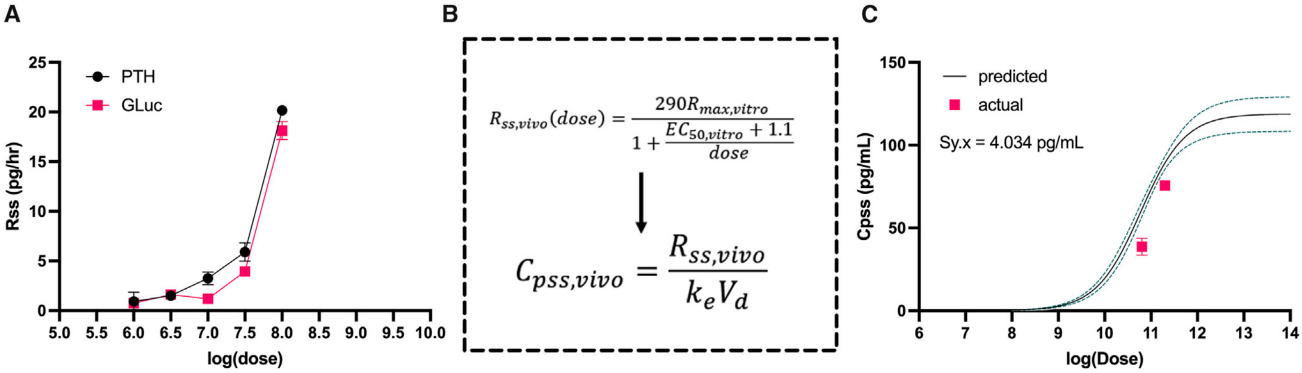

Figure 6. Testing the predictivity of the model using a therapeutic protein

(A) Steady-state secretion rates *in vitro* for parathyroid hormone (PTH) as a function of dose compared with GLuc. (B) Equation from model derivation to predict concentration from steady-state rate. Steady-state rate equation from the *in vivo* scaling model was used along with the elimination rate constant for PTH to predict PTH concentration as a function of dose in mice. (C) Predicted concentrations of PTH with 95% confidence intervals (black line with green dotted line). Pink data points show actual data at two doses which represent mean SD for n = 4 mice/dose. Sum of squares error (Sy.x) shown on graph.

AAV transfection

The AAV-EF1a-GLuc plasmid was cloned using Gateway cloning. Using the same coding sequence for GLuc, the AAV-EF1a-GLuc-WPRE plasmid was created and purchased from VectorBuilder. The packaging plasmids, Rep/Cap and Helper were purchased from Clontech. All plasmid stocks were maxi-prepped using HiPure Maxi column-based preparation kits (Invitrogen) and concentrations as well as purity ratios were recorded using a u-drop plate on VarioScan software (Invitrogen). AAV2 vector was produced using triple-transfection methods in adherent HEK293T cells. Cells were seeded in either 15 cm dishes or Hyperflasks at 40% confluence the day before transfection. Plasmids were mixed at a 1:1:1 molar ratio with PEI and incubated with OptiMEM medium for 15 min. Cell culture medium was replaced with OptiMEM (Gibco) and DNA-PEI complexes were either added dropwise to the culture in 15 cm dishes or directly to the bulk medium for Hyperflasks. Transfection culture was carried out for 96 h until supernatant was collected, filtered through a 0.22 μm PES membrane filter and stored at −80°C. Vector titer was determined by qPCR using an AAV titration kit (Applied Biologic Materials) on Quant Studio 3 (Thermo Fisher).

For the dose and MOI studies, AAV2 vector was purchased from Vector Builder, which supplied aliquots of prepped and purified virus at titers greater than 1×10^{12}. All viral stocks were thawed once prior to *in vitro* transduction to create serial dilutions used for the study. Viral stocks used for *in vivo* studies were thawed immediately prior to use and kept on ice during saline resuspension.

Parameter finding studies

For cellular secretion rate, HepG2 cells were seeded at varying cell density in 12-well plates (10%, 25%, 50%, and 100%). The following day, cells were transduced with AAV2-EF1a-IRES-GFP vector at an MOI of 20,000 vg/cell. Virus-containing medium was incubated with cells for 4 h before medium change. Seventy-two hours post-transduction, cells were perfused with medium at a rate of 0.2 mL/h and supernatant was collected into microfuge tubes at 1-h increments. Secretion rate was measured using a GLuc assay. Transduced cell number was determined by quantifying the number of GFP+ cells with image cytometry (Celigo, Nexcelom).

For route of administration and sex study, male and female mice were injected either intravenously via tail vein, intraperitoneally, or subcutaneously on the dorsal flank with 100 μL viral solution in sterile saline. Each animal received a dose of 2.5×10^9 vg of the AAV2-EF1a-GLuc vector. For initial understanding of dose-dependent kinetics, an additional intravenous group of mice was enrolled at a higher dose of 1×10^{10} vg. At each blood collection time point, mice were briefly warmed using a heating lamp and 100 μL of whole blood was collected in EDTA-coated tubes. During blood collection, tubes were stored at 4°C and immediately spun at 2,000 × *g* for 5 min to isolate plasma.

Single-dose pharmacokinetic study and mass balance

The rate of change of the mass of transgenic protein in the plasma compartment (D) is defined as the secretion rate of the protein into the plasma (R) minus the rate of elimination of the protein (E). This is described in a one-compartment mass balance (Equation 1).

$$\frac{dD}{dt} = R - E. \qquad \text{(Equation 1)}$$

The elimination rate is assumed to be D times the elimination rate constant (k_e), while D equals the plasma concentration (C_p) times the volume of distribution (V_d). Making these substitutions gives the mass in the form of Equation 2.

$$V_d\frac{dC_p}{dt} = R - k_e V_d C_p. \qquad \text{(Equation 2)}$$

To determine k_e and V_d for recombinant GLuc in mice, a pharmacokinetic study was performed using purified rather than secreted

protein (thus, R = 0 in Equation 2). Male mice were randomly assigned to each time point with three mice per group. Mice were injected with a dose of 1 μg of GLuc in 100 μL saline solution via tail vein at 5-min intervals measured using a stopwatch. Blood samples (150 μL) were taken from the opposite tail vein at 5, 15, 30, 60, and 90 min post-injection, using one group at each time point. Heparinized blood collection tubes were used, and plasma was extracted after centrifugation at 2,000 × g for 5 min, after which C_p of GLuc was measured. A single-dose, one-compartment pharmacokinetic model (Equation 3) was derived from Equation 2. SimBiology software (Mathworks) was used to fit Equation 3 to the data to determine k_e V_d for GLuc.

$$Cp(t) = \frac{Dose}{V_d} e^{-k_e t}. \quad \text{(Equation 3)}$$

In vitro secretion assays

HepG2 cells were expanded in complete EMEM and frozen in aliquots for each study run. Cell aliquots were seeded at passage 4 in a six-well plate and allowed to expand for 3 days. One day prior to transduction, cells were passaged at 20,000 cells/well and allowed to adhere overnight in 48-well plates. Each study run included six experimental wells and three controls. Immediately after seeding, cells were counted using bright-field image cytometry (Celigo, Nexcelom) to ensure consistent cell seeding.

The next day, cells were transduced with AAV2-EF1a-GLuc at various doses ranging from 10 to 10^7 vg per well by added 75 μL of viral solution in a total volume of 250 μL complete medium. Four hours later, medium was replaced with 500 μL of complete medium. Each study run included six different doses and was repeated until a total of three replicates were used. The replicate doses were run using different combinations each time. On day 3 post-transduction, cell confluence was measured using bright-field image cytometry to ensure consistent cell density.

To measure the steady-state secretion rate of GLuc, a perfusion culture system developed by our lab was used.[44] In brief, wells were plugged with stoppers with input and output blunt tip needles that supplied fresh medium via syringe pump and collected supernatant in downstream tubing, respectively. A multi-head adapter for a fraction collector was used to collect output streams into microfuge tubes in 90-min intervals. Flow rate was 0.5 mL per hour. Perfusion was run for 18 h, and samples were immediately assayed for GLuc activity. The resulting secretion rates were calculated from the GLuc concentration. Immediately following the perfusion run, cells were stained with Calcein and Hoechst (Nexcelom) to ensure consistent viability post-run.

Transduction efficiency was measured by performing the dose-ranging transductions with AAV2-CMV-GFP viral particles. Three replicates were used for each dose and cells were transduced in 96-well plates at 3,000 cells/well. Transduction efficiency was quantified using bright-field image cytometry to count GFP+ and total cells.

In vivo dose ranging studies

Animals were randomly assigned dosing groups and cage mates received the same dose. Male mice were injected intravenously via tail vein with 200 μL of viral solution containing specified doses of AAV2-EF1a-GLuc ranging from 10^8 to $10^{11.5}$ vg/mouse every half order of magnitude. Negative controls received 200 μL sterile saline solution. At weeks 3 and 5 post-injection, 125 μL whole blood was collected in EDTA-coated tubes to measure steady-state GLuc concentration.

Tissue analysis

Animals were euthanized using CO_2 asphyxiation for 5 min and cardiac puncture was performed immediately via the right ventricle to extract whole blood. The carotid artery was cut and trans-cardiac perfusion was performed with 5 mL sterile saline. Heart, liver, kidney, lung, and spleen samples were extracted and flash-frozen in liquid nitrogen and then stored at −80°C. Prior to freezing, wet weight of the livers was measured. Efficiency of AAV delivery was measured by quantifying the number of AAV genome copies per μg of genomic DNA in the tissue by qPCR. A DNeasy kit was used (QIAGEN) to extract DNA from homogenized samples. qPCR was performed using primers for the ITR region on AAV which were as follows: 5′-GGAACCCCTAGTGATGGAGTT-3′ and 5′-CGGCCTCAGTGAGCGA-3′.

GLuc assay

GLuc, is a naturally secreted form of luciferase that is 19.9 kDa in size. Its native substrate, coelenterazine (CTZ), reacts with the luciferase to emit light at a peak of 480 nm with a broad emission spectrum. This protein has minimal toxicity in rodents and is measurable on a wide dynamic range of over 5 orders of magnitude.[45] In addition, the GLuc reporter can be easily measured within minutes with both a microplate assay and *in vivo* imaging and with higher sensitivity than other reporter molecules.[42]

GLuc concentration was measured using a bioluminescent flash assay. Recombinant GLuc protein (Nanolight Technologies) was used to create serial dilutions from the picogram/mL to microgram/mL range and used as a standard curve with linearity on a wide, dynamic range. The substrate, native CTZ enzyme was reconstituted at 2.7 mg/mL in 200-proof ethanol. Working solutions were made fresh, immediately prior to assay with a 1:1,000 dilution in PBS. Twenty microliters of sample or standard was added to black-walled, clear-bottom 96-well plates and 100 μL of CTZ solution and immediately read using a bioluminescent plate reader (Varioskan, Thermo Fisher). All samples were read forward and in reverse to account for any time-dependent signal degradation. No more than 12 samples were run at a time. The concentration of GLuc in each sample was calculated by taking the average of the forward and reverse readings.

Literature mining

To determine the dose-response relationship between AAV and expression in mice when administered systemically, a literature

search was conducted. Publications that included data that show the concentration or expression after day 21 post-injection were considered at steady state. Publications were only included if at least four different doses were used, and concentration or expression was plotted as a function of dose. Data were extracted from graphs using WebPlot Digitizer. In brief, axes were calibrated and set based on values shown to yield estimated data points on each figure.

Predictivity testing

To test the model with a different protein, AAV2-EF1α-PTH vector was used, which encodes for full-length PTH. As in previous studies, male mice were injected intravenously via tail vein with 200 μL of viral solution containing either $10^{10.8}$ or $10^{11.3}$ vg/mouse. Negative controls received 200 μL sterile saline solution. At weeks 3 and 5 post-injection, 125 μL whole blood was collected in heparin-coated tubes to measure steady-state PTH concentration.

Modeling time-dependent plasma concentration

For the initial parameter finding studies, the GLuc concentrations as a function of time were well-fit by an increasing exponential decay model (Equation 4), which is the solution to Equation 2 for a constant R with β substituted for k_e. The plasma concentration increases from an initial value of zero up to its steady-state concentration (C_{pss}) at a rate governed by a parameter, β.

$$C_p(t) = C_{pss}\left(1 - e^{-\beta t}\right). \qquad \text{(Equation 4)}$$

Hill equation modeling of dose-response curves and scaling *in vitro* to *in vivo*

For the *in vitro* dose-response study, the secretion rate for a given dose was defined as the average of the final six fractions collected from the perfusion culture with that dose. Each *in vivo* dose-response data point, measured as plasma concentrations of protein, was converted to a protein secretion rate using the mass balance to match the units of the *in vitro* data. At the time of measurement, the plasma concentration had reached a steady state ($C_p = C_{pss}$), so its rate of change was zero. Thus, Equation 2 can be rearranged to produce Equation 5, where the secretion rate of the protein into the plasma reached its steady-state value ($R = R_{ss}$). For the *in vivo* dose-response study, the average of the two steady-state concentrations, converted to secretion rates, were used to plot the dose-response curve.

$$R_{ss} = k_e V_d C_{pss}. \qquad \text{(Equation 5)}$$

Dose-response data were plotted as the secretion rate versus log (MOI) for *in vitro* experiments, and secretion rate versus log (dose) for *in vivo* experiments. These plots revealed hyperbolic dose-response curves that are well-fit by the E_{max} model using a Hill coefficient of 1 (Equation 6), defined by the maximum secretion rate (R_{max}) and the dose that produces the EC_{50}. Equation 6 was fit to each dataset using the equivalent four-parameter E_{max} equation in GraphPad Prism, again with the Hill coefficient set to 1, with the "Bottom" parameter set to zero, and a 95% confidence interval.

$$R_{ss}(dose) = \frac{R_{max}}{1 + \frac{EC_{50}}{Dose}}. \qquad \text{(Equation 6)}$$

To scale the *in vitro* dose-response curve to the *in vivo* curve, the fit parameters were compared. It was found that multiplying the R_{max} by one factor, in our specific example ∼290, and adding a second factor to shift the EC_{50} of the *in vitro* curve by ∼1 log order could produce the *in vivo* curve.

Literature data modeling and statistics

Literature data were plotted and fit using a hyperbolic dose-response curve. Predicted versus actual data were plotted for each dataset with a linear trendline. The EC_{50} values estimated from the curve fits were compared for each source and significance was measured using one-way ANOVA ($p < 0.05$). The dose-span was calculated from the difference between the highest and lowest doses tested. The sum of squares for each dataset was determined using the curve-fit analysis and plotted as a function of the log (dose span). A linear trendline was used to determine the correlation between dose span and correlation.

DATA AVAILABILITY

Data related to this work can be made available upon request.

SUPPLEMENTAL INFORMATION

Supplemental information can be found online at https://doi.org/10.1016/j.omtm.2022.10.011.

ACKNOWLEDGMENTS

We thank Justin Gabrielski for providing support of vector production and animal studies. This research was conducted with support under grant nos. R01EB028782 and R01EB012521 awarded by the National Institutes of Health.

AUTHOR CONTRIBUTIONS

Conceptualization, B.P., A.B., C.R., and P.E.; execution of experiments, A.B., K.S., P.E., and R.B.; formal analysis, A.B., P.E., and B.P.; manuscript preparation, A.B., P.E., R.B., C.R., and B.P.; funding acquisition, B.P.

DECLARATION OF INTERESTS

All authors declare no competing interests.

REFERENCES

1. Lau, J.L., and Dunn, M.K. (2018). Therapeutic peptides: historical perspectives, current development trends, and future directions. Bioorg. Med. Chem. *26*, 2700–2707.
2. Ecker, D.M., Jones, S.D., and Levine, H.L. (2015). The therapeutic monoclonal antibody market. mAbs *7*, 9–14.
3. Daugherty, A.L., and Mrsny, R.J. (2006). Formulation and delivery issues for monoclonal antibody therapeutics. Adv. Drug Deliv. Rev. *58*, 686–706.

4. Chowdhury, E.A., Meno-Tetang, G., Chang, H.Y., Wu, S., Huang, H.W., Jamier, T., Chandran, J., and Shah, D.K. (2021). Current progress and limitations of AAV mediated delivery of protein therapeutic genes and the importance of developing quantitative pharmacokinetic/pharmacodynamic (PK/PD) models. Adv. Drug Deliv. Rev. *170*, 214–237.

5. Zane, D., Feldman, P.L., Sawyer, T., Sobol, Z., and Hawes, J. (2021). Development and regulatory challenges for peptide therapeutics. Int. J. Toxicol. *40*, 108–124.

6. Nathwani, A.C., Tuddenham, E.G.D., Rangarajan, S., Rosales, C., McIntosh, J., Linch, D.C., Chowdary, P., Riddell, A., Pie, A.J., Harrington, C., et al. (2011). Adenovirus-associated virus vector-mediated gene transfer in hemophilia B. N. Engl. J. Med. *365*, 2357–2365.

7. Maguire, A.M., Simonelli, F., Pierce, E.A., Pugh, E.N., Jr., Mingozzi, F., Bennicelli, J., Banfi, S., Marshall, K.A., Testa, F., Surace, E.M., et al. (2008). Safety and efficacy of gene transfer for Leber's congenital amaurosis. N. Engl. J. Med. *358*, 2240–2248.

8. Hwu, W.L., Muramatsu, S.i., Tseng, S.H., Tzen, K.Y., Lee, N.C., Chien, Y.H., Snyder, R.O., Byrne, B.J., Tai, C.H., and Wu, R.M. (2012). Gene therapy for aromatic L-amino acid decarboxylase deficiency. Sci. Transl. Med. *4*, 134ra161.

9. Gaudet, D., Méthot, J., Déry, S., Brisson, D., Essiembre, C., Tremblay, G., Tremblay, K., de Wal, J., Twisk, J., van den Bulk, N., et al. (2013). Efficacy and long-term safety of alipogene tiparvovec (AAV1-LPLS447X) gene therapy for lipoprotein lipase deficiency: an open-label trial. Gene Ther. *20*, 361–369.

10. Deverman, B.E., Ravina, B.M., Bankiewicz, K.S., Paul, S.M., and Sah, D.W.Y. (2018). Gene therapy for neurological disorders: progress and prospects. Nat. Rev. Drug Discov. *17*, 641–659.

11. Srivastava, A. (2016). In vivo tissue-tropism of adeno-associated viral vectors. Curr. Opin. Virol. *21*, 75–80.

12. Asokan, A., Schaffer, D.V., and Samulski, R.J. (2012). The AAV vector toolkit: poised at the clinical crossroads. Mol. Ther. *20*, 699–708.

13. Zolotukhin, S., and Vandenberghe, L.H. (2022). AAV capsid design: a Goldilocks challenge. Trends Mol. Med. *28*, 183–193.

14. Gao, G., Lu, Y., Calcedo, R., Grant, R.L., Bell, P., Wang, L., Figueredo, J., Lock, M., and Wilson, J.M. (2006). Biology of AAV serotype vectors in liver-directed gene transfer to nonhuman primates. Mol. Ther. *13*, 77–87.

15. Domenger, C., and Grimm, D. (2019). Next-generation AAV vectors-do not judge a virus (only) by its cover. Hum. Mol. Genet. *28*, 3–14.

16. Schuettrumpf, J., Herzog, R.W., Schlachterman, A., Kaufhold, A., Stafford, D.W., and Arruda, V.R. (2005). Factor IX variants improve gene therapy efficacy for hemophilia B. Blood *105*, 2316–2323.

17. Monahan, P.E., Sun, J., Gui, T., Hu, G., Hannah, W.B., Wichlan, D.G., Wu, Z., Grieger, J.C., Li, C., Suwanmanee, T., et al. (2015). Employing a gain-of-function factor IX variant R338L to advance the efficacy and safety of hemophilia B human gene therapy: preclinical evaluation supporting an ongoing adeno-associated virus clinical trial. Hum. Gene Ther. *26*, 69–81.

18. Wu, Z., Sun, J., Zhang, T., Yin, C., Yin, F., Van Dyke, T., Samulski, R.J., and Monahan, P.E. (2008). Optimization of self-complementary AAV vectors for liver-directed expression results in sustained correction of hemophilia B at low vector dose. Mol. Ther. *16*, 280–289.

19. Lisowski, L., Dane, A.P., Chu, K., Zhang, Y., Cunningham, S.C., Wilson, E.M., Nygaard, S., Grompe, M., Alexander, I.E., and Kay, M.A. (2014). Selection and evaluation of clinically relevant AAV variants in a xenograft liver model. Nature *506*, 382–386.

20. Herkt, M., and Thum, T. (2021). Pharmacokinetics and proceedings in clinical application of nucleic acid therapeutics. Mol. Ther. *29*, 521–539.

21. Yu, R.Z., Grundy, J.S., Henry, S.P., Kim, T.W., Norris, D.A., Burkey, J., Wang, Y., Vick, A., and Geary, R.S. (2015). Predictive dose-based estimation of systemic exposure multiples in mouse and monkey relative to human for antisense oligonucleotides with 2′-o-(2-methoxyethyl) modifications. Mol. Ther. Nucleic Acids *4*, e218.

22. Shemesh, C.S., Hsu, J.C., Hosseini, I., Shen, B.Q., Rotte, A., Twomey, P., Girish, S., and Wu, B. (2021). Personalized cancer vaccines: clinical landscape, challenges, and opportunities. Mol. Ther. *29*, 555–570.

23. Adachi, K., Klocke, K., Roy, P., Rodricks, D., Chapman, M.S., and Nakai, H. (2015). 37. Capacity of viral genome packaging and internal volumes of AAV viral particles. Mol. Ther. *23*, S17.

24. Belov, A., Schultz, K., Forshee, R., and Tegenge, M.A. (2021). Opportunities and challenges for applying model-informed drug development approaches to gene therapies. CPT Pharmacometrics Syst. Pharmacol. *10* (4), 286–290.

25. Aksenov, S., Roberts, J.C., Mugundu, G., Mueller, K.T., Bhattacharya, I., and Tortorici, M.A. (2021). Current and next steps toward prediction of human dose for gene therapy using translational dose-response studies. Clin. Pharmacol. Ther. *110*, 1176–1179.

26. Tang, F., Wong, H., and Ng, C.M. (2021). Rational clinical dose selection of adeno-associated virus-mediated gene therapy based on allometric principles. Clin. Pharmacol. Ther. *110*, 803–807.

27. Nathwani, A.C., Cochrane, M., McIntosh, J., Ng, C.Y.C., Zhou, J., Gray, J.T., and Davidoff, A.M. (2009). Enhancing transduction of the liver by adeno-associated viral vectors. Gene Ther. *16*, 60–69.

28. Nathwani, A.C., Gray, J.T., Ng, C.Y.C., Zhou, J., Spence, Y., Waddington, S.N., Tuddenham, E.G.D., Kemball-Cook, G., McIntosh, J., Boon-Spijker, M., et al. (2006). Self-complementary adeno-associated virus vectors containing a novel liver-specific human factor IX expression cassette enable highly efficient transduction of murine and nonhuman primate liver. Blood *107*, 2653–2661.

29. Davidoff, A.M., Gray, J.T., Ng, C.Y.C., Zhang, Y., Zhou, J., Spence, Y., Bakar, Y., and Nathwani, A.C. (2005). Comparison of the ability of adeno-associated viral vectors pseudotyped with serotype 2, 5, and 8 capsid proteins to mediate efficient transduction of the liver in murine and nonhuman primate models. Mol. Ther. *11*, 875–888.

30. Grimm, D., Zhou, S., Nakai, H., Thomas, C.E., Storm, T.A., Fuess, S., Matsushita, T., Allen, J., Surosky, R., Lochrie, M., et al. (2003). Preclinical in vivo evaluation of pseudotyped adeno-associated virus vectors for liver gene therapy. Blood *102*, 2412–2419.

31. Nakai, H., Fuess, S., Storm, T.A., Muramatsu, S.i., Nara, Y., and Kay, M.A. (2005). Unrestricted hepatocyte transduction with adeno-associated virus serotype 8 vectors in mice. J. Virol. *79*, 214–224.

32. Nakai, H., Thomas, C.E., Storm, T.A., Fuess, S., Powell, S., Wright, J.F., and Kay, M.A. (2002). A limited number of transducible hepatocytes restricts a wide-range linear vector dose response in recombinant adeno-associated virus-mediated liver transduction. J. Virol. *76*, 11343–11349.

33. Fröhlich, F., Reiser, A., Fink, L., Woschée, D., Ligon, T., Theis, F.J., Rädler, J.O., and Hasenauer, J. (2019). Multi-experiment nonlinear mixed effect modeling of single-cell translation kinetics after transfection. NPJ Syst. Biol. Appl. *5*, 1.

34. Ellis, B.L., Hirsch, M.L., Barker, J.C., Connelly, J.P., Steininger, R.J., 3rd, and Porteus, M.H. (2013). A survey of ex vivo/in vitro transduction efficiency of mammalian primary cells and cell lines with Nine natural adeno-associated virus (AAV1-9) and one engineered adeno-associated virus serotype. Virol. J. *10*, 74.

35. Zhong, L., Li, W., Li, Y., Zhao, W., Wu, J., Li, B., Maina, N., Bischof, D., Qing, K., Weigel-Kelley, K.A., et al. (2006). Evaluation of primitive murine hematopoietic stem and progenitor cell transduction in vitro and in vivo by recombinant adeno-associated virus vector serotypes 1 through 5. Hum. Gene Ther. *17*, 321–333.

36. Rajman, I. (2008). PK/PD modelling and simulations: utility in drug development. Drug Discov. Today *13*, 341–346.

37. Marsic, D., Méndez-Gómez, H.R., and Zolotukhin, S. (2015). High-accuracy biodistribution analysis of adeno-associated virus variants by double barcode sequencing. Mol. Ther. Methods Clin. Dev. *2*, 15041.

38. Davidoff, A.M., Ng, C.Y.C., Zhou, J., Spence, Y., and Nathwani, A.C. (2003). Sex significantly influences transduction of murine liver by recombinant adeno-associated viral vectors through an androgen-dependent pathway. Blood *102*, 480–488.

39. Ogura, T., Mizukami, H., Mimuro, J., Madoiwa, S., Okada, T., Matsushita, T., Urabe, M., Kume, A., Hamada, H., Yoshikawa, H., et al. (2006). Utility of intraperitoneal administration as a route of AAV serotype 5 vector-mediated neonatal gene transfer. J. Gene Med. *8*, 990–997.

40. Mingozzi, F., Schüttrumpf, J., Arruda, V.R., Liu, Y., Liu, Y.L., High, K.A., Xiao, W., and Herzog, R.W. (2002). Improved hepatic gene transfer by using an adeno-associated virus serotype 5 vector. J. Virol. *76*, 10497–10502.

41. Dane, A.P., Wowro, S.J., Cunningham, S.C., and Alexander, I.E. (2013). Comparison of gene transfer to the murine liver following intraperitoneal and intraportal delivery of hepatotropic AAV pseudo-serotypes. Gene Ther. *20*, 460–464.

42. Tannous, B.A., and Teng, J. (2011). Secreted blood reporters: insights and applications. Biotechnol. Adv. *29*, 997–1003.

43. Leiker, A.J., Yen, T.W.F., Eastwood, D.C., Doffek, K.M., Szabo, A., Evans, D.B., and Wang, T.S. (2013). Factors that influence parathyroid hormone half-life: determining if new intraoperative criteria are needed. JAMA Surg. *148*, 602–606.

44. Erickson, P., Houwayek, T., Burr, A., Teryek, M., and Parekkadan, B. (2021). A continuous flow cell culture system for precision cell stimulation and time-resolved profiling of cell secretion. Anal. Biochem. *625*, 114213.

45. Tamayo, A.G., Shukor, S., Burr, A., Erickson, P., and Parekkadan, B. (2020). Tracking leukemic T-cell transcriptional dynamics in vivo with a blood-based reporter assay. FEBS Open Bio. *10*, 1868–1879.

OMTM, Volume *27*

Supplemental information

Allometric-like scaling of AAV gene therapy for systemic protein delivery

Alexandra Burr, Patrick Erickson, Raphaela Bento, Kariman Shama, Charles Roth, and Biju Parekkadan

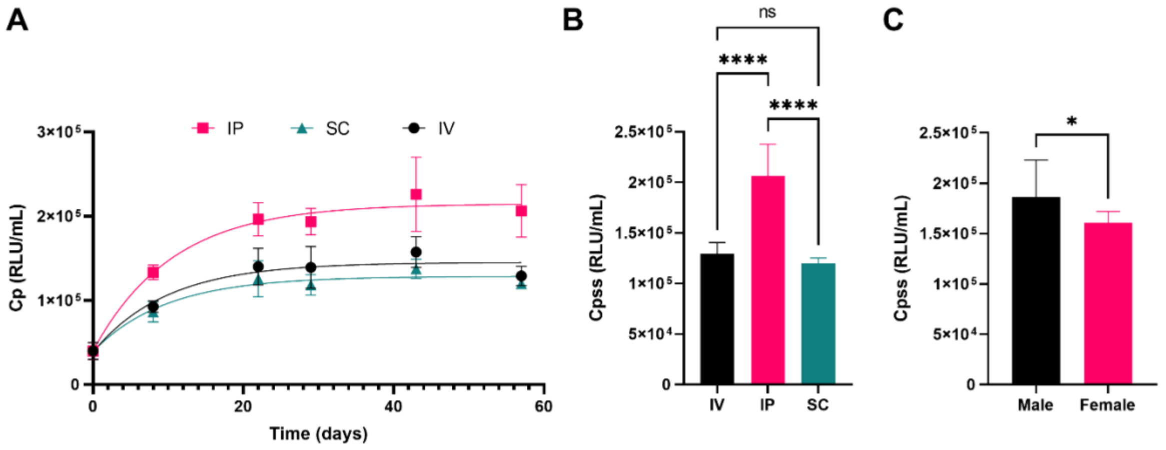

Figure S1. GLuc concentration varies with systemic administration route. (A) Concentration of GLuc in the plasma (Cp) over time (RLU/mL) for each administration route: IV = intravenous, IP = intraperitoneal, SC = subcutaneous. Data points show are the mean ± SD for replicates (n=8, 4 male/4 female, per group). Solid lines represent curve-fit for an exponential plateau equation to estimate steady-state. (B) Steady-state concentration (RLU/mL) as determined by the curve-fit is shown for each administration route. (C) Comparison of male and female concentrations for all routes of administration. Bars represent mean ± SD for replicates. NS = non-significant, *p<0.05, **** p<0.0001.

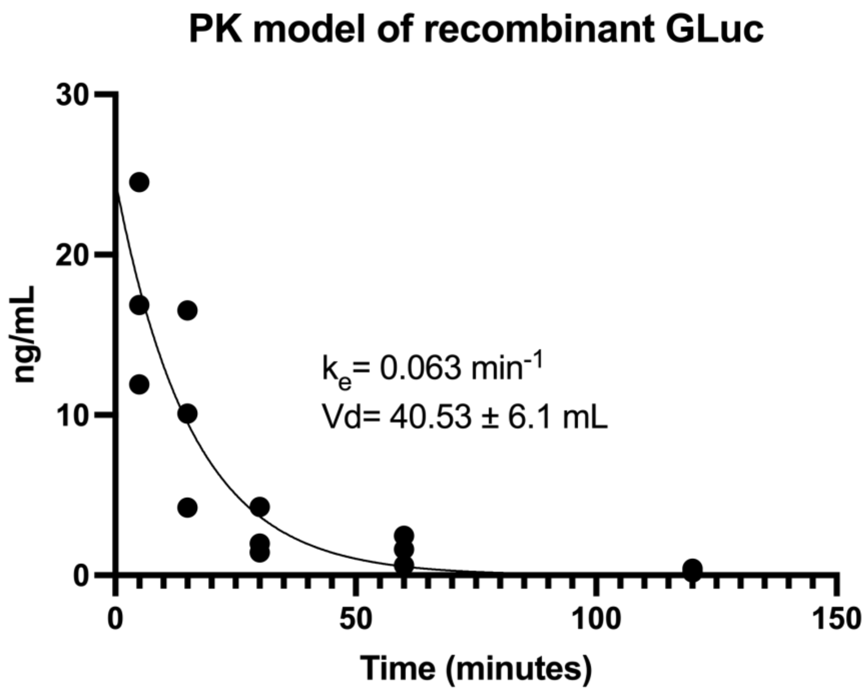

Figure S2. A single dose kinetic study with 1ug of the recombinant Gluc protein injected intravenously (n=3 mice/timepoint). Parameters for GLuc were determined by fitting the data to Equation 3 and extracting values of the elimination constant and the volume of distribution.

Table S1. Study information for data sets used for literature mining study

Publication	Serotype	Promoter / Transgene	# of doses tested	# of mice	Route of Administration	Sex, Strain	Timepoint for dose-response
Nathwani et al, 2009	scAAV2/8	LP1-hFIX	4	5-8	tail vein IV	male C57BL/6	57 days
	scAAV2/9						
	scAAV2/7						
	scAAV2/rh10						
Nathwani et al, 2006	scAAV2/8	LP1-hFIXco	4	4	tail vein IV	male C57BL/6	42 days
	ssAAV2/8	HCR-hAAT-hFIX	6				
Davidoff et al, 2005	ssAAV5/5	HCR-hAAT-hFIX	8	4	tail vein IV	male C57BL/6	116 days
	ssAAV2/8		6				
Grimm et al, 2003	ssAAV2/2	HCR-hAAT-hFIX	6	4-5	portal vein	C57BL/6	42 days
	ssAAV2/1		5				
	ssAAV2/6		7				
Nakai et al, 2002	ssAAV2/2	HCR-hAAT-hFIX	9	5	portal vein	female C57BL/6, male C57BL/6 rag-1	70 days
Nakai et al, 2005	ssAAV2/1	EF1α-nlslacZ	4	3-6	portal vein	male C57BL/6	42 days
	ssAAV2/2						
	ssAAV2/8						

Abbreviation: Ss: single-stranded genome, sc: self-complementary stranded genome
